# COVID-19: Technology, Social Connections, Loneliness, and Leisure Activities: An International Study Protocol

**DOI:** 10.3389/fsoc.2020.574811

**Published:** 2020-11-19

**Authors:** Hannah R. Marston, Loredana Ivan, Mireia Fernández-Ardèvol, Andrea Rosales Climent, Madelin Gómez-León, Daniel Blanche-T, Sarah Earle, Pei-Chun Ko, Sophie Colas, Burcu Bilir, Halime Öztürk Çalikoglu, Hasan Arslan, Rubal Kanozia, Ulla Kriebernegg, Franziska Großschädl, Felix Reer, Thorsten Quandt, Sandra C. Buttigieg, Paula Alexandra Silva, Vera Gallistl, Rebekka Rohner

**Affiliations:** ^1^Health and Wellbeing Strategic Research Area, The Open University, Milton Keynes, United Kingdom; ^2^Communication Department, The National University of Political Studies and Public Administration (SNSPA), Bucharest, Romania; ^3^Communication Networks & Social Change (CNSC) Research Group, Internet Interdisciplinary Institute (IN3) Research Institute, Universitat Oberta de Catalunya, Catalonia, Spain; ^4^Centre for University Core, Singapore University of Social Science, Singapore, Singapore; ^5^Le Centre de Recherches Individus, Épreuves, Sociétés (CeRIES), University of Lille, Lille, France; ^6^Institute for Anthropological Research in Africa (IARA), KU Leuven, Belgium; ^7^Graduate School of Educational Sciences, Canakkale Onsekiz Mart University, Canakkale, Turkey; ^8^Department of Mass Communication and Media Studies, Central University of Punjab, Bathinda, India; ^9^Age and Care Research Group Graz, University of Graz, Graz, Austria; ^10^Institute of Nursing Science and Age and Care Research Group, Medical University Graz, Graz, Austria; ^11^Department of Communication, University of Munster, Munster, Germany; ^12^Department of Health Sciences Management, Faculty of Health Sciences, University of Malta, Msida, Malta; ^13^Department of Informatics Engineering (DEI), Centre for Informatics and Systems (CISUC), Faculty of Science and Technology, University of Coimbra, Coimbra, Portugal; ^14^Ageing, Generations, Life-Course Research Group, Department of Sociology, University of Vienna, Vienna, Austria

**Keywords:** gerontology, pandemic (COVID-19), international rapid response, quantitative data, social media, gerontechnology, social science research

## Abstract

Drawn from the stress process model, the pandemic has imposed substantial stress to individual economic and mental well-being and has brought unprecedented disruptions to social life. In light of social distancing measures, and in particular physical distancing because of lockdown policies, the use of digital technologies has been regarded as the alternative to maintain economic and social activities. This paper aims to describe the design and implementation of an online survey created as an urgent, international response to the COVID-19 pandemic. The online survey described here responds to the need of understanding the effects of the pandemic on social interactions/relations and to provide findings on the extent to which digital technology is being utilized by citizens across different communities and countries around the world. It also aims to analyze the association of use of digital technologies with psychological well-being and levels of loneliness. The data will be based on the ongoing survey (comprised of several existing and validated instruments on digital use, psychological well-being and loneliness), open for 3 months after roll out (ends September) across 11 countries (Austria, France, Germany, India, Malta, Portugal, Romania, Spain, Turkey, and UK). Participants include residents aged 18 years and older in the countries and snowball sampling is employed via social media platforms. We anticipate that the findings of the survey will provide useful and much needed information on the prevalence of use and intensities of digital technologies among different age groups, gender, socioeconomic groups in a comparative perspective. Moreover, we expect that the future analysis of the data collected will show that different types of digital technologies and intensities of use are associated with psychological well-being and loneliness. To conclude, these findings from the study are expected to bring in our understanding the role of digital technologies in affecting individual social and emotional connections during a crisis.

## Introduction

The ongoing COVID-19 pandemic, is one of the worst pandemics in human history and in the last 100 years, on a global scale and has resulted in ~21,294,845 (267,291—in the last 24 h) infected cases, and 761,799 deaths (5,985—in the last 24 h) (World Health Organization, 2020) with the statistics still on the rise. The pandemic has caused disruptions across the usual social and economic activities. Reducing physical contact, social gatherings and the complete lockdown have indirectly redefined the common practice of work, caregiving, support and social interaction.

To date, there is a growing body of scholarly research relating to the impact of COVID-19 and citizens across different countries and continents. This includes Ammar et al. (2020a,b,c) who deployed an online survey in seven languages to understand how this pandemic was impacting on the daily living and lives of citizens in relation to social distancing, isolation and home confinement. Furthermore, Bentlage et al. (2020) and Chtourou et al. (2020) who are members of the same project have explored and provided practical recommendations for maintaining an active lifestyle and physical activity during the pandemic. Such recommendations include exergaming, yoga, and home-based exercise, with appropriate amounts of intensity conducted for both adults and children.

Scholarly research surrounding the use, benefits and impacts of digital technology in the lives of older citizens, as well as those of younger citizens (Ito et al., 2010; Cotten et al., 2014; Marston, 2019) has been growing over the last 30+ years (Czaja and Barr, 1989). The application of digital technologies in influencing social and psychological well-being have been widely studied with the focus of the types of digital connections and the intensities of using digital technologies among general population (Rosenfeld and Thomas, 2012; Hofstra et al., 2017; Verduyn et al., 2017; Rafalow, 2018; Henwood and Marent, 2019; Shah et al., 2019). The demographic profiles of the users, such as age, gender and education are important factors to understand the accessibility, frequencies and types of digital use in creating and enhancing social connections and support.

When focusing on social isolation, loneliness and the digital divide, there is a myriad of scholarly research surrounding older adults and includes exploring older adults' attitudes toward technology adoption and use (Mitzner et al., 2010; Marston, 2012; O'brien et al., 2012; Fernández-Ardèvol and Ivan, 2015; Marston et al., 2016, 2019; Rosales and Fernández-Ardèvol, 2016a,b; Fernández-Ardèvol et al., 2017; Rosales et al., 2018; Fernández-Ardèvol, 2020), leisure activities (Genoe et al., 2018), supporting independence, social support and connectedness while reducing loneliness (Bouma et al., 2004; Cotten et al., 2013; Czaja et al., 2018; Schlomann et al., 2020), the digital divide and inequalities (Cotten et al., 2009; Gilleard et al., 2015; Lagacé et al., 2015; Friemel, 2016; Hargittai and Dobransky, 2017; Ball et al., 2019; Fernández-Ardèvol, 2019), telemedicine and emerging technologies for healthcare (Sixsmith and Sixsmith, 2000; Mitzner et al., 2012; Czaja et al., 2013; Sharit et al., 2019), and to support age-in-place (Mynatt et al., 2000, 2004; Beer et al., 2012; Marston and van Hoof, 2019; White et al., 2020).

Based on the growing literature highlighted above and in the next section, coupled with the rise of digital technologies in terms of social networking sites, virtual conferencing, etc. there is alternative thoughts and approaches to maintaining social connections and activities during the crisis. In general, this growth in scholarly research illustrates a fast-moving arena within the fields of sociology, general social sciences, computer science and gerontechnology. In addition to a series of national and international research projects focusing on the various impacts and roles that technology can play within society (Ivan et al., Accepted; Ivan and Hebblethwaite, 2016; Loos et al., 2018, 2019; Marston et al., 2019; Nimrod, 2019; Nimrod and Ivan, 2019; Gallistl and Nimrod, 2020).

The purpose and rationale of this paper is to describe a study protocol which includes a description of the Consortium members, the online instrument used for data collection, coupled with future work and dissemination activities. This study protocol details the urgency and the international response to the COVID-19 pandemic. We anticipate the preliminary findings will provide an insight into the use of digital technologies and the impacts of using digital technologies on psychological well-being and loneliness.

## Background and Literature Review

### Interdisciplinary Research Projects

In what follows we summarize previous projects that have targeted individuals through using online tools to collect data across different countries and individual's age-groups.

The ACT (aging + communication + technologies) project (ACT project, 2014–2021) is a Canadian-funded partnership that brings more than 45 international researchers, community partners and institutions together. By means of different pilot projects and case studies, ACT aims to explore and understand the transformational experiences of aging through various mediums of communication. It develops research in three main areas.

First, “Agency in Aging” encompasses a program of research that involves individuals and communities in the development of participatory action research projects in the field of the digital arts that have both scholarly and creative outcomes. Second, “Critical Mediations” examines the everyday life practices, the various mediated experiences of adults in later life, and the existing cultures of aging. Finally, “Telecommunication Technologies” investigates aging in the context of networked societies. Of interest to this paper are two cross-country projects, one which consists of an online longitudinal study about older audiences in the digital media environments and another, Grannies on the Net, about the role of information and communication technologies (ICTs) in grandmothers' interactions with close and distant social ties.

The online longitudinal research about older audiences in the digital media environments (2016–2020) (Loos et al., 2018, 2019) has revealed the rather diverse media practices (both digital and analog) and the heterogeneity of older online technology users among the six countries which are part of the project. This project underlines the importance of cross-national analysis when we talk about information communication technology (ICT) use by different generations. In analyzing data, researchers not only focused on the relation between well-being and media-based leisure of older adults (Gallistl and Nimrod, 2020) and on technology use by different generations of older adults (Ivan et al., Accepted), but also on media-displacement (Nimrod, 2019)–a process by which traditional media has been replaced to a more or lesser extent by the new media, in the way people communicate, get information, and solve everyday tasks.

The results of the Grannies on the Net project, which examines the role of ICTs in grandmothers' interactions with close and distant social ties, uncovered the varying motives and use strategies grandmothers deploy to communicate with a diversity of actors while revealing commonalities in the challenges they face in different cultural contexts. We tackled the role of ICT in reducing grandmothers' feeling of loneliness and their need to share everyday experiences with loved ones (Ivan and Hebblethwaite, 2016). Also, we analyzed the role ICT plays in older women's leisure (Nimrod and Ivan, 2019). On the one hand, it helps older women to remain active and socially engaged by saving time that could be used for their leisure, facilitating participation in various activities and allowing for a more meaningful leisure experiences both online and offline. On the other hand, ICT can often entail wasted time and disrupted involvement, or simply served as a “time filler.” The authors of this respective study conclude how the impact of ICT may vary across various age cohorts and gender, which in turn may impact the various leisure activities by the respective participants.

The Technology In Later Life (TILL) project (2015–2017) is a multi-centered, international study comprising of two countries (UK and Canada) and four sites: two rural (South Wales, UK McBride, BC) and two urban (Milton Keynes, UK and Regina, SK). The TILL study aimed to explore the use, perceptions and impacts of technology on adults aged 70+ years residing in these four geographic locations. The findings from the TILL study ascertained two main overarching themes. The first is “facilitators of technology”, which relates to the sharing of information by the respective participants, and for those participants who do use technology, the extent to which this afforded them a sense of security. The second theme is “detractors of technology”, which identified a sense of apprehension of using technology. Recommendations from the TILL study proposed the notion of promoting technology based on the strengths and positive opportunities to facilitate health and well-being. Secondly, a peer support network(s) should be considered and created to assist novice users in understanding how to use ICTs facilitated by experienced peer users.

The notion of exploring and understanding how intergenerational relationships can be enhanced and maintained via technology use was also suggested (Marston et al., 2019). From a leisure standpoint, Genoe et al. (2018) identified technology as a primary means of accessing leisure activities such as games, hobbies and maintaining social connections. However, those participants did note challenges including, difficulty in using and updating software, concerns surrounding privacy and security and their overall lack of confidence and interest. Although technology may facilitate leisure activities and engagement from these respective participants, to overcome the issues and drawbacks identified by these participants may be afforded through educational/community opportunities.

Finally, intergenerational experiences were noted through data collection in the TILL study and were pivotal in continuing social connections with family and friends (Freeman et al., 2020). For example, findings showed older adults leveraged existing friendships and familial relationships when learning and adjusting to new digital devices and technologies. This is particularly the case when geographic distance is playing an integral role in these respective intergenerational relationships. The notion of a “digital gathering place” is motivated and implemented by all familial relationships and friends to ensure communication is continued.

The Technology 4 Young Adults (Technology 4 Young Adults (T4YA) Project, 2017) was a pilot study to understand the perception, use and impact technology has on young adults aged 18–34 years—the Millennials—in the UK. Findings from the T4YA initial study identified several primary themes including privacy issues and concerns, activities relating to content and sharing of information confidence, usability, and functionality of using technology and associated platforms coupled with various day-to-day activities (Marston, 2019).

The interdisciplinary research project “Internet and Mental Health” (2016–2019) aimed to investigate the psychosocial effects of using online media. In Germany, a nationally representative survey of 1,929 adolescents and younger adults (aged 14–39 years) focused on topics such as social media and gaming disorder (Reer et al., 2020; Tang et al., 2020a), the fear of missing out (Reer et al., 2019), or sexual harassment in online contexts (Tang et al., 2020b). A primary goal of this project was to examine how the use of ICTs is associated with different indicators of mental health, loneliness, depression, and anxiety. Findings from this project underline the central role of the Internet in the daily lives of the younger generation and emphasize its relevance for younger user's psychosocial well-being.

The “Being Connected at Home - Making use of digital devices in later life” project (BCONNECT HOME, 2018–2020) investigates fundamental changes in the contemporary experience of later life, at the intersection of digital infrastructures, place and the experience of “being connected” (Fernández-Ardèvol et al., 2019). It addresses a research gap by exploring and theorizing the role of digital communication devices—such as smartphones (that have been tracked), tablets, PCs, apps, fitness trackers, pedometers, or “brain games” —in relation to the modern life course. It combines this theoretical approach with a practical goal through co-design by involving diverse older people and other relevant stakeholders in “Academic Work Places” in the Netherlands, Spain, Sweden and Canada. The project is contextualized by debates around age in place, loneliness and social isolation, and the idea that these are age-related challenges that require interventions.

The ACCESS Project (Supporting Digital Literacy and Appropriation of ICT by older people, 2018–2021) aims to provide and evaluate socially embedded learning opportunities for older adults who are digitally excluded. The aim of the project is to support older adults to learn and appropriate new digital technologies later in life in Austria, Germany, Italy, Finland and Japan. It addresses a research gap by exploring informal, non-formal and formal learning settings in later life and further developing it through combination of such approaches with different forms of learning (courses, senior-to-senior approaches, praxlabs) (Gallistl et al., 2020).

The project “App-Solute News: Intergenerational Learning, Digitalisation and the Media” (2020–2022) looks at the daily routine of newspaper reading in the context of the transition from analog (printed paper) to digital (e-paper and app). Intergenerational teams of students and adults 60+ in the region of Styria in Austria will compare their reading habits, work with printed papers and e-paper apps, and create digital stories about their experiences. The aim is to investigate the narratives that are formed in these intergenerational encounters with regard to the transition from analog to digital. The project aims at understanding the role of age and age-related stereotypes in digitalisation processes.

Building upon the extensive projects conducted before, this study is expected to bring in a sociological lens to look into the influence of using digital technologies. The ongoing COVID-19 pandemic as a health crisis has resulted in the disruption of work, family support, education and social interaction. These changes may have formed heath, social and economic stressors to one's psychological well-being in the times of ongoing crisis. The stress-process model illustrates how economic strains and changing social circumstances become long-term stressors that impact individual health and well-being and how various sociodemographic groups may have different degrees of impacts (Pearlin et al., 2005). This framework provides a new perspective to look into the prevalence of how individuals from different sociodemographic backgrounds change to use the digital technologies and also provides explanations on the extent digital technologies influence individual well-being and loneliness to cope with the external pandemic.

While the aforementioned projects address issues related to the use and impact and appropriation of digital technology in people's lives, this paper reports on a study that, although addressing adjacent goals to those of the projects above, emerges in the context of the current COVID-19 pandemic scenario. Freeman et al. (2020) continue to note the importance of intergenerational support and communication, is having and has been pivotal since the start of the pandemic. Whereby, the use of various forms of digital technologies (e.g., communication tools and social media platforms) as a primary method of maintaining intergenerational social connectedness and support has been key. For example, for those older adults who do not have internet access, while their children or grandchildren who do have internet, has afforded the older person to be able to receive groceries during the strict lockdown(s) period. During the lockdown period of the pandemic and especially for those citizens in society categorized as vulnerable and who needed to shield, having access to the internet was key to ensuring day-to-day/monthly supplies of groceries as well as maintaining communication and leisure activities. The survey deployed in this study has and will capture the various activities of individual respondents and their communities during the pandemic. We anticipate we will be able to report findings surrounding what type of leisure activities have been conducted, the various community efforts employed (especially aimed at the vulnerable populations), and the health and well-being of respondents relating to social connection and loneliness.

## Methods

The project- COVID-19: Technology, Social Connections, Loneliness and Leisure Activities (2020a) has employed two theoretical theories, firstly a life course perspective (Elder, 1985; Green, 2017; Hutchinson, 2018). Taking a life course perspective will afford the Consortium to analyse the collected data to specific personal, and historical life events, forming a “personal biography” (Elder, 1985). This project is not primarily aimed at older adults but adults who are 18 years or older and given this unprecedented pandemic has impacted the lives of all citizens globally. Furthermore, taking a life course perspective will provide a quantifiable understanding of how digital technologies have been used by citizens and their respective experiences pre pandemic as well as during the pandemic.

Secondly, ecology theory will afford the Consortium to examine, explore and discuss the role played by digital technologies during the pandemic, as the medium to improve citizens' social connections, which in turn enables citizens social resources and support to reduce feeling of loneliness. Additionally, the ecology theory will facilitate application associated to 1. the levels of loneliness, 2. the decomposition of the user profile and, 3. to ascertain the types of digital technology used by citizens during the pandemic across four areas: 1. Individual, 2. Relationship (e.g., family, peers, and friends), 3. Community (e.g., groups, networks, workplace, neighborhoods), and 4. Societal (Berkes and Folke, 1998; Foxon et al., 2009; Smith and Stirling, 2010; Anderies, 2014; McPhearson et al., 2016; Ahlborg et al., 2019).

### Aims and Objectives

The aim of this paper is to describe the online instrument of the COVID-19: Technology, Social Connections, Loneliness and Leisure Activities (2020a,b). This online survey explores how digital technology was used, accessed, perceived and impacted the lives of citizens across 11 countries (UK, Malta, France, Germany, Austria, Romania, India, Singapore, Portugal, Spain, and Turkey) and 10 languages (English, German, French, Hindi, Mandarin, Portuguese, Romanian, Turkish, Spanish, Catalan). This will provide useful insights on the use of digital technologies and the impact of use on loneliness from the surveys across 11 countries.

The surveys across 11 countries under the project: COVID-19: Technology, Social Connections, Loneliness and Leisure Activities (2020a) have the following overarching objectives:

- explore the behavior and use of technology by citizens during the COVID-19 pandemic
- explore how citizens use technology to connect with COVID-19 support groups
- explore how citizens use technology to share information during the COVID-19 pandemic
- explore the health and well-being of citizens during COVID-19 relating to loneliness and digital health literacy
- explore the perception and notion of a national emergency alert system by citizens
- explore the behavior and narratives of users who are using technology to maintain familial and friendship social connections and build new connections during the COVID-19 pandemic.

As can be seen, these aims are incorporated into the online survey and will provide important data on how people have been using digital technologies and the differentiations of digital technologies during the pandemic. Moreover, the survey also covered health-related questions, which enable researchers to look into the health and well-being of the respondents.

### Ethics

Ethical approval for conducting the online survey was initially granted by the lead and respective University, The Open University (HREC/3551/MARSTON) located in the UK.

Subsequent ethical approval has been processed and approved by the National University of Political Studies and Public Administration (SNSPA–Romania), Open University of Catalonia (Spain), Singapore University of Social Sciences (Singapore), Department of Health Sciences Management, University of Malta–(Malta), the Department of Informatics Engineering (DEI)/Center for Informatics and Systems (CISUC) at the University of Coimbra (Portugal), the Department of Mass Communication and Media Studies at the Central University of Punjab (India), Nursing Science, Age and Care Research Group at the Medical University Graz (Austria), Department of Sociology at the University of Vienna, the Department of Age and Care Research Group at the University of Graz (Austria), the Department of Communication at the University of Münster (Germany), and Canakkale Onsekiz Mart University in Turkey.

All versions of the survey, study information sheet and certification from respective Universities have been shared with The Open University Human Research Ethics Committee (HREC) to ensure those respective records are maintained and kept up to date.

All documentation lists the ethical approval granted by the lead University and the respective University ethical committee or board. Informed consent was obtained by all participants taking part in this online survey.

### Partner Recruitment

Upon completion and deployment of the English version of the survey, the project lead (HRM) contacted colleagues in a bid to expand the survey and increase participant recruitment. This resulted in the lead for Romania (RO) requesting the word documents to be translated and rolled out across Romania.

This, in turn, led to the UK and RO leads utilizing their existing networks and inviting their respective colleagues to join the project. HRM provided a description of the study, responsibilities, and expectations to prospective partners, and once a partner confirmed their involvement, the English survey and study information sheet was provided to the respective partner to allow for back/translation to commence. A copy of the ethics application by HRM at The Open University was shared with the respective partner to facilitate the respective partner to expedite their own ethical approval process.

### Participant Recruitment

Given the focus of this work and the restrictions imposed by the respective Universities and Countries, participant recruitment is being conducted through multiple channels in what constitutes a *non-probabilistic* sampling process, usual in online research (Ayhan, 2011). This includes social media channels (e.g., Facebook, LinkedIn, Twitter, WhatsApp), existing mailing lists, stakeholder organizations (e.g., Age Northern Ireland). Subjects aged 18 years and older are allowed to take part in the online survey.

To obtain insights into the current COVID-19 health crisis during the lockdown period, a virtual *snowball sampling technique* is applied through the project team's networks to build a sample quickly and across different countries. This technique is increasingly applied given the facilities that the Internet opens to investigate phenomena in current societies (Benfield and Szlemko, 2006; Baltar and Brunet, 2012). This approach has advantages such as reaching individuals faster (hence minimizing the period of collecting and processing data), expanding the sample size and the scope of the study across different settings (e.g., different countries) given the flexibility to apply them in different formats and languages. Nevertheless, this approach also has some shortcomings, such as *selection bias* related to the online population reached and the non-representativeness of the sample to the general population, aspects that should be considered when analyzing the data. Additionally, India chose to recruit participants using an alternative approach to the other countries, and includes using a direct WhatsApp link, followed by frequent follow-ups. The approach was deemed by the respective partner as a means of encouraging prospective participants to participate in this survey. Furthermore, this approach provides a more personalized approach in helping to get good numbers of responses within an Indian context.

Currently, some surveys are still ongoing, and it is not possible to provide a complete overview of participant demographics of this study. However, the 11 study sites involved in the project include an interesting subset of countries, representing different socio-economic groups and population characteristics (Table 1). This will lend the project and the collected data to include and report diversity. Furthermore, this will provide insight concerning countries of high, middle, and low-income, surrounding different demographic characteristics, for example population densities, median age, life expectancy, etc. Overall, the countries involved in this study represent ~22% of the world share of the population.

**Table 1:** Demographic characteristics of the countries included in this study (United Nations, (2019); The World Bank Group, (n.d.); Worldometer, (n.d.-a), (n.d.-b)).

**Country**	**Total population**	**Population density/Km^**2**^**	**Median age**	**Life expectancy at birth**	**World share of population**	**Economic group**
Austria	9,006,398	109	43	82.05	0.12%	High-Income
France	65,273,511	119	42	83.13	0.84%	High-Income
Germany	83,783,942	240	46	81.88	1.07%	High-Income
India	1,380,004,385	464	28	70.42	17.70%	Lower-Middle Income
Malta	441,543	1380	43	83.06	0.01%	High-Income
Portugal	10,196,709	111	46	82.65	0.13%	High-Income
Romania	19,237,691	84	43	76.50	0.25%	High-Income
Singapore	5,850,342	8358	42	86.15	0.08%	High-Income
Spain	46,754,778	94	45	83.99	0.60%	High-Income
Turkey	84,339,067	110	32	78.45	1.08%	Upper-Middle-Income
United Kingdom	67,886,011	281	40	81.77	0.87%	High-Income

### Online Survey

The online survey rolled out for the study uses the Qualtrics platform as the sole method of deployment across different networks. The English version of the survey is based on previous iterations and studies conducted by the lead author (Marston, 2012, 2019; Marston et al., 2016, 2019). During the design of the English version of the survey, revisions were conducted to the instrument to meet the aims and objectives of the study.

For a new survey to be added to the Qualtrics platform, a copy is made within the platform and the lead author transfers (manually, copy and paste) the translated version of the survey into the new project. The project is named in that respective language. Once the translation is transferred, the survey is exported into Microsoft Word, saved, and shared with the partner(s) to review for any errors, changes in questions to reflect the cultural context and resent for amendments within the Qualtrics platform. In some instances back to HRM, the survey may have several colleagues from one institution reviewing the documentation and suggesting revisions. Once the respective partners have agreed that the documentation is correct, the respective online measuring instrument goes through some usability testing, on various hardware devices, and changes are suggested. Once this stage has been agreed, the instrument is copied again, and republished, in preparation for rollout.

### Survey Languages and Translated Versions

Table 2 presents the versions of the survey, translated languages and the lead for each country. At present there are 10 countries which currently have the survey deployed and staggered rollout dates because of ethical approval. Survey versions by additional partners will be open for 3 months respectively upon ethical approval and rollout.

**Table 2:** Displays the survey iterations.

**#**	**Lead**	**Country**	**Language**	**Countries deployed**	**Survey deployment**	**Ethical approval**
1	Hannah R. Marston, Sarah Earle	UK	English	USA, Canada, Singapore, Australia	03.04.2020	Approved
2	Loredana Ivan	Romania (RO)	Romanian	Romania	20.04.2020	Approved
3	Mireia Fernández-Ardèvo, D. Blanche Tarragó, A. Rosales Climent, M. Gomez Leon	Spain	Catalan Spanish	Spain and Hispanic America	04.05.2020	Approved
4	Sophie Colas	France	French	France, Switzerland, Belgium, Quebec (CA)	12.05.2020	Approved via The Open University
5	Pei-Chun Ko	Singapore	Mandarin	Singapore	12.05.2020	Approved
6	Halime Öztürk Çalikoǧlu, Hasan Arslan, Burcu Bilir	Turkey	Turkish	Turkey	26.06.2020	Approved
7	Rubal Kanozia	India	Hindi	India	31.05.2020	Approved
8	Vera Gallistl, Ulla Kriebernegg, Franziska Groβschädl, Gerhilde Schüttengruber, Rebekka Rohner, Hanna Kottl	Austria	German	Germany,Switzerland	05.06.2020	Approved
9	Feliz Reer, Thorsten Quandt	Germany	German	Austria/Switzerland	04.06.2020	Approved
10	Sandra C. Buttigieg	Malta	British English	Malta	19.05.2020	Approved
11	Paula Alexandra Silva	Portugal	European Portuguese	Portugal	29.05.2020	Approved

### Measures

The surveys build on previous iterations from the Technology 4 Younger Adults (T4YA) study (Marston, 2019), the iStoppFalls EU project (Marston et al., 2016), the Technology In Later Life (TILL) project (Genoe et al., 2018; Marston et al., 2019; Freeman et al., 2020), and the doctoral work of HRM (Marston, 2012).

In total there have been seven scholarly works published using earlier iterations of this survey (Marston, 2012, 2019; Marston et al., 2016, 2019; Genoe et al., 2018; Freeman et al., 2020). An earlier iteration of a survey deployed in the TILL study is available for download (Marston et al., 2019). The previous version of the survey deployed in the TILL study comprised an 80-item survey.

This new iteration of the English version of the survey comprises 65-items (Table 3). For some of the languages/countries there have been some minor alterations to the survey to represent each country's respective culture, laws, and educational systems. For example, the Singapore arm requested the questions relating to sexuality should be deleted for both the English and Mandarin versions which would be rolled out across the respective networks. The consideration is due to the fact that Singapore still retains the section 377A of the Penal Code (Singapore Statues Online, 2020). Though the law is not enforced (Chen, 2013), the partner for the Singapore arm decided to revise the question about the sexuality to a conventional question of the participants' gender with an option where participants can fill out the identity freely. The French partner also requested the questions on sexual orientation and transgender identity to be removed due to its too intimate nature.

**Table 3:** Presents an overview of the measuring variables included in the COVID-19 technology, self-isolation, health, well-being, and leisure activities study.

**Survey section**	**Survey items**	**Example of question(s)**	**Source**
Participant Consent Q1	Participant consent	NA	NA
Section A Q2–Q16	Computer ownership, purpose of using a computer, length and frequency of use, video game console ownership, favorite type of game to play. How the internet is accessed, frequency of using the internet, purpose of using the internet. Type of social media platforms used, frequency and purpose of using social media platforms.	• Have you used a computer? (select answer) • How long have you used a computer for? (select answer) • Do you use social networking sites • Why do you use social media sites	(Marston, 2012, 2019; Marston et al., 2016, 2019; Genoe et al., 2018; Freeman et al., 2020)
Section B Q17–35	COVID-19 related questions: purpose of using technology/internet/social media platforms to share information, communicate with support groups, challenges faced during COVID-19.	• Since COVID-19, have you become a member of a community support group on social media (e.g., Facebook or similar)? • Since COVID-19, have you signed up to receive community deliveries/assistance (e.g., groceries from a local newsagents /community shop / butcher's / farmer's market)? • Please describe why you WOULD sign up to receive community deliveries and/or assistance. If you choose not to answer please write N/A • Please describe why you are unsure or you WOULD NOT sign up to receive community deliveries and/or assistance. If you choose not to answer please write N/A • Since COVID-19, how have you been spending your time? Select all that apply • My death and that of people close to me (e.g., family members) • Limited social contact with friends and family • Not having a job to go back to • I am on my own. I have no support (e.g., no children etc.) • Education for my child/children • On your digital device do you share information about COVID-19? • Why do you share information surrounding COVID-19–select all that apply	Q17-25 - new items added aimed specifically at Covid-19 experiences. Q26-35 - adapted from (Genoe et al., 2018; Marston, 2019; Marston et al., 2019; Freeman et al., 2020)
Section C Q36–Q40	13-items, 1–7 pt Likert COVID-19 related questions	• Making new social connections/friendships • Spending more time with spouse/family members • Being a key worker and helping in this pandemic • Being isolated more • Giving something back • Please describe how your communication behavior(s) has changed since COVID-19. If you choose not to answer please write N/A • Since COVID-19, has the way you use your digital devices changed your way of communication?	New items added relating to Covid-19
Section D Q41	18-item measure, 1–7 pt Likert Psychological well-being	• “I am good at managing the responsibilities of daily life.” • “I think it is important to have new experiences that challenge how I think about myself and the world.” • “I judge myself by what I think is important, not by the values of what others think is important.”	Ryff and Keyes, 1995; Ryff and Singer, 1998
Section E Q42	8-item measures, 1–5 pt Likert scale eHealth/digital literacy	• I know where to find helpful health resources on the Internet • I know how to use the health information I find on the Internet to help me • I feel confident in using information from the Internet to make health decisions	Norman and Skinner, 2006
Section F Q43	UCLA Loneliness scale Version 3, 20-item measure, 1-4 pt Likert scale	• How often do you feel that you are “in tune” with the people around you? • How often do you feel part of a group of friends? • How often do you feel that your interests and ideas are not shared by those around you? • How often do you feel that no one really knows you well?	Russell, 1996
Section G Q44–Q49	Social networks, virtual assistants and emergency alert systems	• During the COVID-19 pandemic, how frequently have you communicated with members of your social network? Please rate your answers below on each of the sliders • Does your country have a National Emergency Alert System (e.g., mobile app or SMS)? • Please describe what your thoughts are relating to a National Emergency Alert System (e.g., mobile app or SMS)? If you choose not to answer please write N/A • Virtual Assistants such as Alexa, Google Home, Siri have various features. Please describe whether you think such devices or similar would be suitable as a way of sharing National Emergency Alert Systems. If you choose not to answer please write N/A	New items added to the survey.
Section H Q50–Q65	Demographic questions: gender, age, country, ethnicity, geographic location, education, sexuality, marital status, #of people living your home, #of children living your home, employment status, disability, self-isolation	• Do you meet the criteria for being vulnerable or extremely vulnerable? • Have you had to self-isolate because of COVID-19?	(UK Government, (n.d.); Marston, 2012, 2019; Patten, 2015; Marston et al., 2016, 2019; Genoe et al., 2018; Settlement Hierarchy, 2019; Thrive, 2019; Education in the United States, 2020; Freeman et al., 2020)

Furthermore, a slight change in wording for questions relating to the national emergency alert system were renamed to “Public Warning System” in Singapore. Both versions in India (English and Hindi) as well as the version in European Portuguese, French and German do not include the question relating to ethnicity. In some instances, the types of educational qualification were altered to represent the respective cultural contexts. This occurred for translations in Catalan and Spanish, European Portuguese, and German. The Spanish version was also aimed at rolling out in extended networks across Hispanic America. Additionally, in both the Catalan and Spanish versions the questions relating to a national emergency alert system were deleted, and this was on the guidance of the partners in the project, because they felt that in their targeted countries there were either no such systems in place, or no public discussion on the matter, making the question difficult to answer in most instances.

### Procedure

The project lead for the UK shares a copy of the study information sheet and a copy of the English survey with the new consortium partner. If a partner has additional colleagues, communication is limited to reduce the risk of information being misunderstood and to ensure all communication between the two coordinators is correct. This is a practical approach during the survey translation(s) and testing phases.

Each new country/partner applies for ethical approval through their respective University ethics committee. Additional documentation from the UK partner is shared based on the favorable opinion granted by the Open University (HREC/3551/MARSTON). A copy of the ethics application is also shared to assist the respective partner in completing their ethics application. Furthermore, each partner has the option to attach this document and the favorable opinion to their respective application.

The Qualtrics platform is used to create and deploy the survey to adhere to the Open University policies. All translated surveys from each study partner are transferred into a new project within the platform and exported to Microsoft Word for checking by the Consortium partner. Each revision within the platform is published and allows the consortium partner to test their respective survey for user experience (UX)/usability issues and formatting. At each revision, the consortium partner receives an updated version of the survey via a Word document and is able to review the survey online.

### Survey Deployment

Once ethical approval is granted, the survey was “published” via the Qualtrics platform, which created an independent link that is shared via various channels (specific email(s), anonymous link etc.). The COVID-19: Technology, Social Connections, Loneliness and Leisure Activities (2020b) project website is the portal for all participants to review the study, download the respective study information sheet, and access the online survey.

**Figure 1:**
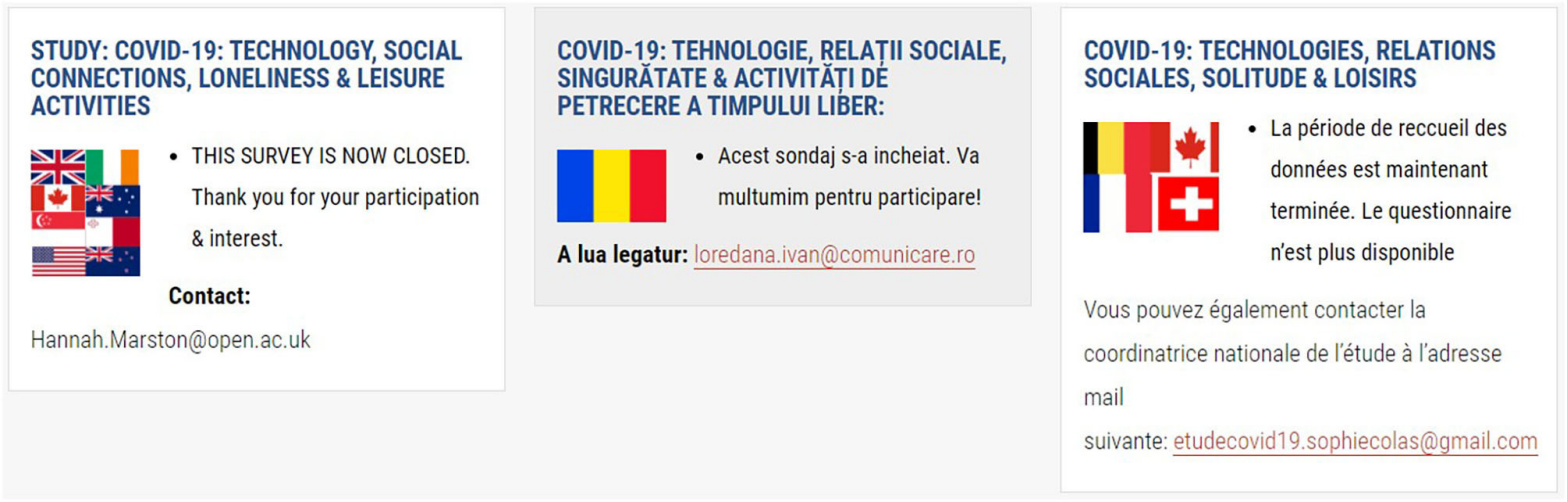
Figure displays three partners involved in the study and their respective flags, study information, contact information. For each partner/country, there were additional links to the online survey, study information sheet, and consent form.

**Figure 2:**
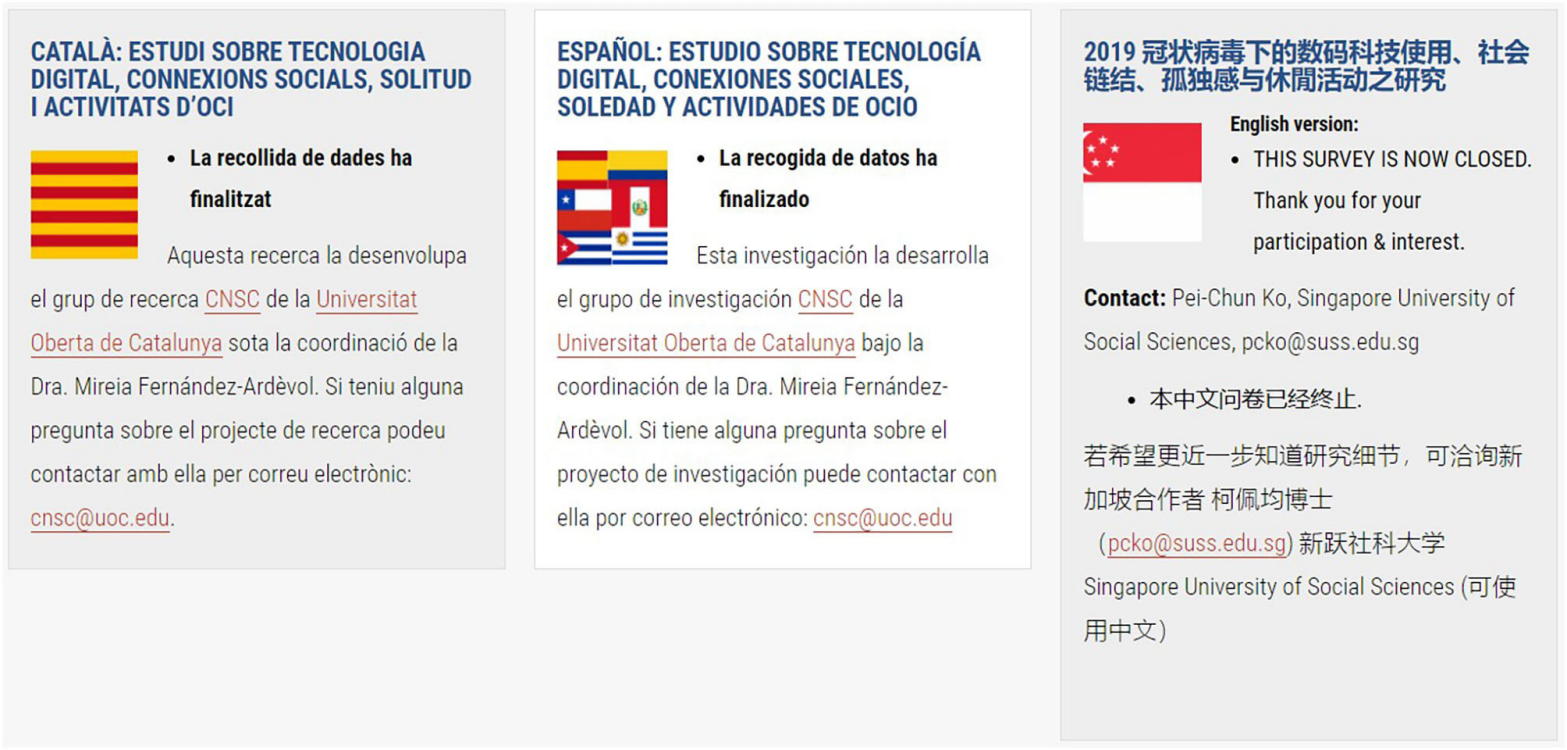
Figure displays three partners involved in the study and their respective flags, study information, contact information. For each partner/country, there were additional links to the online survey, study information sheet, and consent form.

**Figure 3:**
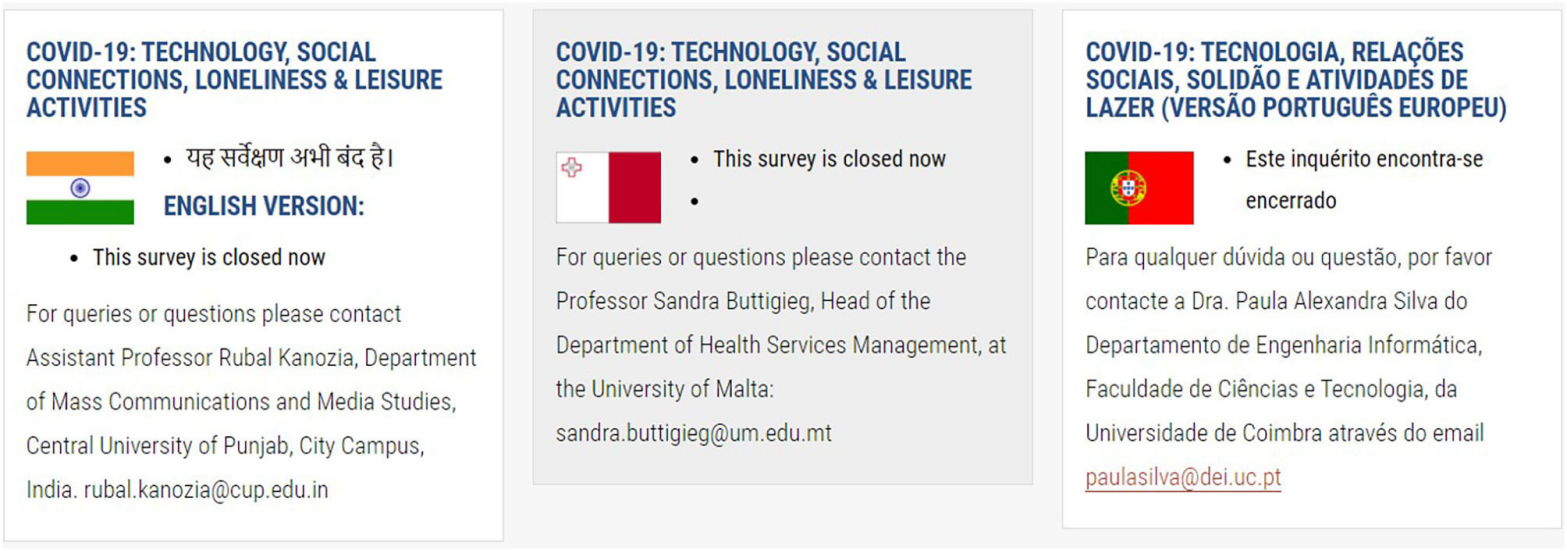
Figure displays three partners involved in the study and their respective flags, study information, contact information. For each partner/country, there were additional links to the online survey, study information sheet, and consent form.

**Figure 4:**
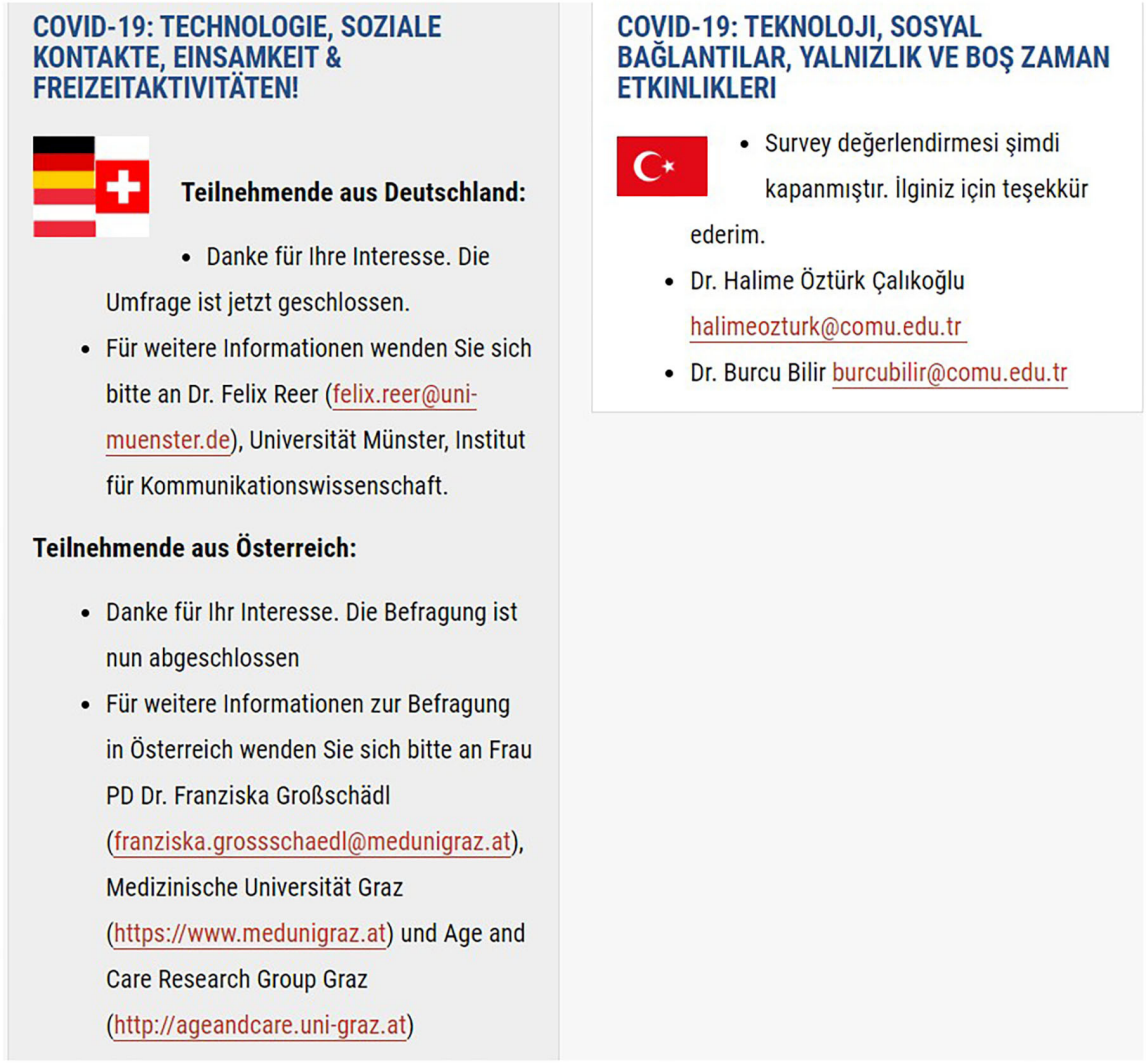
Figure displays three partners involved in the study and their respective flags, study information, contact information. For each partner/country, there were additional links to the online survey, study information sheet, and consent form.

To assist prospective participants to identify the correct survey to complete, a series of flags are placed at the side of each translated section (Figures 1–4). To ensure access to the study information sheet and the respective online survey—there are links in the respective language which highlights the links— “Study Information Sheet” and “Complete Online Survey Here”. The size of the flags helps identify the desired version of the survey. However, an issue that might prevent participants to reach their survey is that the webpage is only available in English.

For each country/consortium partner, there is a contact email address available on the study website and the study information sheet. This facilitates the participant(s) to contact the researcher in charge of the survey in each particular language (Figures 1–4).

### Data Analysis

In this section of the study protocol, we outline how the collected data and subsequent analysis will drive the results forward. Each survey translation closes at various times and at present between 4th July and 29th September 2020. The data analysis will take the form of descriptive statistics with crosstabulations and multivariate regression models to provide preliminary descriptive findings. This in turn will drive the results to afford the Consortium members to explore and understand the collected data from the standpoint of (a) age, (b) gender, (c) education, and (d) country. *T*-tests will be employed to test the differences in the levels of well-being and loneliness feelings among different social groups based on sociodemographic characteristics. Lastly, inferential statistical analysis will be conducted to examine the relationships between digital use and well-being and/or loneliness feelings, with the considerations of different sociodemographic, family structure, and country settings (especially related to COVID-19 measures) of respondents.

Our sample is a convenience sample in all countries included in the study and we do not claim to have statistical and national representative at the country level. Still, we will conduct further analysis if the structure of our sample in each country is comparable with the structure of the adult population with Internet access (age, gender, education level and economic status).

To consider the differences across the countries and cultures included in the study, this project relies on a research team that consists of native speakers and local researchers, who are and will play an important role in contextualizing the respective data. Additionally, members of the research team have the role in comparing the data across the main socio-demographics (age, gender, level of education, economic status) and to provide valuable information regarding the comparability of the sample in the current proposal with the structure of the total adult population in the respective countries.

We anticipate the collected data will provide an insight into the prevalence of different types of digital technologies with the crosstabulations of various sociodemographic characteristics. While each study site will be conducting their own data analysis, we will also be merging the different data sets into STATA to facilitate greater statistical analysis via multivariate analyses to understand the impact(s) of digital technologies on well-being and loneliness. All surveys will be closed by the end of September.

### Online Repository

As part of the ethical process at The Open University an online repository is used—Open Research Data Online (ORDO). A separate project has been created by the lead, and all partners have been invited to join the project. ORDO will be used to store all final documents, data and associated information relating to this study. Each country/region has its individual folder to facilitate ease of organization, access and following the policies of The Open University.

### The Consortium

This is a cooperative project that responds to the need of urgent information during the unexpected COVID-19 pandemic. The project lead has already defined the operational dynamics of the study to grant fast and sustainable outputs (academic journals, policy recommendations) as well as other dynamics in search of the establishment of a consortium able to create an intellectual community around the gathered data. Each study site has worked with the project lead to ensure the translated survey has conducted backwards translation of the online survey and to ensure the meaning(s) of the survey questions are not lost. Each site lead was responsible for their own participant recruitment—via mailing lists, various social media channels (e.g., Twitter and Facebook) and will be responsible for data analysis.

## Discussion and Future Work

In this study protocol we are describing how this international, multi-centered project has been designed to explore and understand how digital technology and associated platforms are being used by citizens and how digital technology impacts on their day-to-day lives during the COVID-19 pandemic, across different countries, regions and cultures. With this in mind, participants of the study are welcome to complete a survey if they are over 18 years old. Given the unprecedented crisis on a global scale, citizens in society are living and experiencing life differently to what society was prior to the pandemic and also differently depending on the country they live in. Therefore, it is important to explore how technology is having an impact on citizens from the age of 18+ years, rather than just focusing on older adults as this will allow for comparisons between generations.

In particular, it is essential to capture the possibilities that digital technology affords citizens during this pandemic a as way of garnering insights with the view to working toward respective pathways to impact, which in turn has the ability to inform policy and decision makers at local, regional, national and international levels. The fact that this survey is being deployed across several different languages, affords the Consortium members to reach out and understand different cultures and demographics. Furthermore, this project lends itself to understanding the necessary multidimensional perspective required to develop effective and meaningful pathways associated to social science and behavioral research, public engagement, knowledge translation and pathways to impact in the future. Moreover, this international project affords the Consortium partners to understand how digital technology is used and impacts society at a time when social distancing measures were varied and may afford the research team to understand and explore possible suggestions and solutions to feed into policy.

This study protocol describes the COVID-19: Technology, Social Connection, Loneliness and Leisure Activities project and we anticipate the findings driven from the collected data will contribute to the fields of gerontechnology, Human Computer Interaction (HCI), gerontology, and social sciences by the creation of a Master dataset and individual datasets from each site. Furthermore, this research will contribute to the existing scholarly research (Morris et al., 2014; Baker et al., 2018; Barbosa Neves et al., 2019; Ammar et al., 2020a,b,c; Fakoya et al., 2020; Marston and Morgan, 2020; Marston et al., 2020; Seifert et al., 2020). The Consortium anticipates the findings from the collected data will indicate how digital technology and associated platforms impact the lives of citizens across various sites during the pandemic. This will afford researchers, stakeholders and policy makers the opportunity in the future to learn and understand how citizens in the respective countries utilized digital technologies during unprecedented times. Additionally, the Consortium will be undertaking a wide variety of public engagement and knowledge translation activities, in addition to ensuring traditional academic outputs are achieved. Members of the Consortium will seek out opportunities to share findings with respective countries at local, regional and national levels of policy in a bid to offer insights and solutions in the future.

Currently, existing scholarly research demonstrates the growing interest in contemporary academe surrounding the use of digital technology as a bridge to maintain social connections and interactions while reducing loneliness; with the aim of understanding how technology has impacted the lives of many citizens worldwide. This study protocol describes how this particular study, to the knowledge of the Consortium, is the first to take an international, interdisciplinary rapid response to the COVID-19 pandemic, while contributing to existing respective disciplines.

The social impacts of the pandemic can be understood through the lens of the digital divide with the intersectionality of education, gender and age. The project is expected to bring in first-hand data to understand the prevalence of technology associated to the different socio-demographic groups. This in turn will afford us to understand whether the COVID-19 pandemic narrows the digital gap or widens it. Incorporated with the loneliness scale and the questions of the activities, researchers can examine the extent to which digital technology use influences individuals' mental well-being and social connections, and the heterogeneity among social groups.

Future work surrounding the Consortium members and the collected data will explore funding opportunities to enable the next phase of this project—pathways to impact and dissemination activities. The former may include an Open Educational Resource (OER) developed and accessible via The Open University which will include input from all project partners and will include content related to the findings. This in turn will enable citizens, policy makers, stakeholders, and researchers on a global scale to access, learn, and understand how digital technologies played a role and impacted the lives of citizens across 11 countries. Dissemination activities will include traditional academic outputs (e.g., journal papers and conference presentations), seminars, booklets/reports and where appropriate contributing to policy.

## Ethics Statement

The studies involving human participants were reviewed and approved by The Open University (UK) HREC/3551/MARSTON. Written informed consent to participate in this study was provided by the participants' legal guardian/next of kin. All study sites received ethical approval prior to survey deployment. The French version of the online survey was approved via the Open University.

## Author Contributions

HM: conceptualization. HM, LI, MF-A, AR, MG-L, DB-T, SE, P-CK, SC, BB, HÖ, HA, RK, UK, FG, FR, TQ, SB, PS, VG, RR, and the COVID19 Technology Consortium Group: writing – original draft preparation, review, and editing. All authors contributed to the article and approved the submitted version.

## Conflict of Interest

The authors declare that the research was conducted in the absence of any commercial or financial relationships that could be construed as a potential conflict of interest.
